# Using microRNAs Networks to Understand Pancreatic Cancer—A Literature Review

**DOI:** 10.3390/biomedicines12081713

**Published:** 2024-08-01

**Authors:** Oskar Przybyszewski, Michał Mik, Michał Nowicki, Michał Kusiński, Melania Mikołajczyk-Solińska, Agnieszka Śliwińska

**Affiliations:** 1 Department of Nucleic Acid Biochemistry, Medical University of Lodz, 251 Pomorska St., 92-213 Lodz, Poland; 2 Department of General and Colorectal Surgery, Medical University of Lodz, 113 Stefana Żeromskiego St., 90-549 Lodz, Poland; michal.mik@umed.lodz.pl (M.M.); michal.edward.nowicki@umed.lodz.pl (M.N.); 3 Department of Endocrinological, General and Oncological Surgery, Medical University of Lodz, 62 Pabianicka St., 93-513 Lodz, Poland; michal.kusinski@umed.lodz.pl; 4 Department of Internal Medicine, Diabetology and Clinical Pharmacology, Medical University of Lodz, 251 Pomorska St., 92-213 Lodz, Poland; melania.mikolajczyk@umed.lodz.pl

**Keywords:** microRNA (miRNA), pancreatic cancer, signaling pathways, proliferation, apoptosis, PDAC, cell cycle

## Abstract

Pancreatic cancer is a severe disease, challenging to diagnose and treat, and thereby characterized by a poor prognosis and a high mortality rate. Pancreatic ductal adenocarcinoma (PDAC) represents approximately 90% of pancreatic cancer cases, while other cases include neuroendocrine carcinoma. Despite the growing knowledge of the pathophysiology of this cancer, the mortality rate caused by it has not been effectively reduced. Recently, microRNAs have aroused great interest among scientists and clinicians, as they are negative regulators of gene expression, which participate in many processes, including those related to the development of pancreatic cancer. The aim of this review is to show how microRNAs (miRNAs) affect key signaling pathways and related cellular processes in pancreatic cancer development, progression, diagnosis and treatment. We included the results of in vitro studies, animal model of pancreatic cancer and those performed on blood, saliva and tumor tissue isolated from patients suffering from PDAC. Our investigation identified numerous dysregulated miRNAs involved in KRAS, JAK/STAT, PI3/AKT, Wnt/β-catenin and TGF-β signaling pathways participating in cell cycle control, proliferation, differentiation, apoptosis and metastasis. Moreover, some miRNAs (miRNA-23a, miRNA-24, miRNA-29c, miRNA-216a) seem to be engaged in a crosstalk between signaling pathways. Evidence concerning the utility of microRNAs in the diagnosis and therapy of this cancer is poor. Therefore, despite growing knowledge of the involvement of miRNAs in several processes associated with pancreatic cancer, we are beginning to recognize and understand their role and usefulness in clinical practice.

## 1. Introduction

In 2019, the incidence of pancreatic cancer diagnoses in the European Union (EU) was 109,000 cases, with a projected increase to 147,000 by 2039 [1]. Pancreatic ductal adenocarcinoma (PDAC) accounts for approximately 90% of these cases, originating from the epithelial cells lining the pancreatic ducts, while the remainder are neuroendocrine tumors arising from the pancreatic endocrine tissue [2,3,4]. The disease’s high mortality rate is predominantly attributable to delayed diagnosis, as patients often remain asymptomatic until the cancer reaches an advanced stage [1]. As of 2022, the median survival postdiagnosis is approximately 11 months [1].

Currently, the diagnosis of pancreatic cancer relies primarily on imaging modalities such as magnetic resonance imaging (MRI), computed tomography (CT) or endoscopic ultrasound (EUS). However, there remains a critical deficiency in effective biological markers for early detection of asymptomatic pancreatic lesions. The widely used marker CA 19-9 lacks the requisite sensitive and specific for pancreatic cancer diagnosis [5,6,7,8]. Unfortunately, approximately 50% of patients present with metastasis at the time of diagnosis. Surgical resection, when feasible, followed by chemotherapy and/or radiotherapy constitutes the standard therapeutic approach, with neoadjuvant treatment recommended for advanced stage patients. Chemotherapeutic agents like gemcitabine, often combined with fluorouracil, oxaliplatin and irinotecan, are commonly employed, yet the 5-year survival rate for PDAC patients remains low, ranging from 3% to 6% [2,9,10]. Therefore, the efficacy of this approach is very limited, and, in emergency cases, only palliative treatment is available. Given these challenges, there is an urgent imperative to identify novel biomarkers for early detection of pancreatic lesions in asymptomatic individuals for future screening purposes [9,10,11,12,13,14,15].

MicroRNAs (miRNAs) play a crucial role in regulating gene expression at the mRNA level, including genes involved in the dysregulation of signaling pathways associated with cancer such as KRAS, JAK/STAT, PI3/AKT, Wnt/β-catenin and TGF-β. These pathways govern critical cellular processes such as cell cycle control, proliferation, differentiation, and apoptosis, which are frequently dysregulated in cancer. While the specific roles of miRNAs in these processes are still under investigation, their intricate interplay with multiple signaling pathways remains complex and incompletely understood. Therefore, the aim of this review is to elucidate the roles of miRNAs in pathways relevant to pancreatic tumorigenesis.

## 2. Materials and Methods

We searched for relevant papers in: PubMed and Google Scholar, using the following keywords: miRNA, pancreatic cancer, PDAC, pancreatic cancer epidemiology, pancreatic cancer, potential therapeutic targets, synthesis of miRNA, PI3K/AKT signaling pathway, KRAS signaling pathway, JAK/STAT signaling pathway, TGF-β signaling pathway, Wnt/β-Catenin signaling pathway, cell cycle, proliferation, apoptosis, metastasis of pancreatic cancer, migration of pancreatic cancer, diagnosis of pancreatic cancer, development of PDAC. We traced studies carried out on both cell models (in vitro) and animal models (in vivo) and research involving human samples isolated from patients suffering from pancreatic cancer or pancreatitis. We included papers published between 1993 and 2022.

## 3. Results

### 3.1. Epidemiology and Development of Pancreatic Cancer

Pancreatic cancer stands out as one of the most aggressive malignancies globally. In 2017, the incidence of new cases worldwide reached 447,665. Over the past 25 years, both incidence and mortality rates have increased significantly by 55% and 53%, respectively. Pancreatic cancer accounts for 1.8% of all cancers cases and contributes to 4.6% cancer-related deaths [1]. In the European Union (including the UK) the number of new cases rose from 59,000 in 1990 to 109,000 in 2019, with projections suggesting a doubling of deaths by 2060 compared to 2019 data [1,9]. Late diagnosis is primarily responsible for the high mortality rates associated with pancreatic cancer [1,16], with approximately 50% of patients diagnosed at an advanced metastatic stage, correlating with a dismal 5-year survival rate of approximately 9.7% [1,17]. There are no significant gender disparities in incidence and mortality rates of pancreatic cancer. The disease exhibits a consistent age-related pattern, with incidence progressively increasing after the age of 30, and peaking after the age of 80. Sociodemographic factors significantly influence the epidemiological burden [18,19]. Established risk factors for pancreatic cancer include cigarette smoking, excessive alcohol consumption, high coffee and fat intake, obesity, diabetes mellitus, chronic pancreatitis, familial predisposition [2,11]. The majority of pancreatic cancers are concentrated in the Asia-Pacific region, with fewer occurrences reported in the Middle East and North Africa [1].

**Figure 1:**
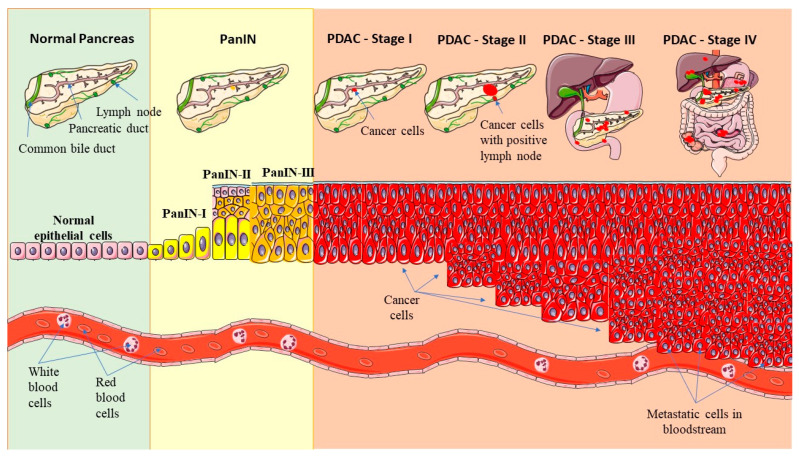
The stages of PDAC development, including the progression of precursor lesions called pancreatic intraepithelial neoplasia (PanIN). The figure was partly generated using Servier Medical Art, provided by Servier, licensed under a Creative Commons Attribution 3.0 unported license.

Approximately 90% of pancreatic cancers are categorized as exocrine tumors, while the remainder originate from the endocrine part of the organ. The progression of PDAC is illustrated in Figure 1. The endocrine portion of the pancreas constitutes only 1% of its total mass of the organ and consists of clusters of cells known as Langerhans islands. These islets produce hormones such as insulin, glucagon, somatostatin and pancreatic polypeptide, which are released into the bloodstream [20]. The exocrine part of the pancreas is composed of acinar cells that synthesize digestive proenzymes. These enzymes are subsequently secreted into pancreatic juice, which enters the gastrointestinal tract lumen through the pancreatic duct [20].

PDAC evolves from precursor lesions called pancreatic intraepithelial neoplasia or PanIN. PanIN stratified based on cytological atypia into: PanIN-I (low-grade dysplasia), PanIN-II (moderate dysplasia) or PanIN-III (high-grade dysplasia). Initiation of PanIN formation involves dysregulation of critical signaling pathways and processes including DNA damage response (TP53 (tumor protein p53)), cell cycle regulation (CDKN2A (cyclin-dependent kinase inhibitor 2A)—act as tumor suppressors by regulating inhibition of the cell cycle) and transforming growth factor β (TGFβ) signaling mediated by SMAD4 (SMAD4 (suppressor of mothers against decapentaplegic 4)). Dysfunctions in these processes coincide with aberrant activation of Hedgehog signaling, Wnt/β-catetnin signaling, integrin signaling, JAK/STAT signaling, KRAS signaling, homophilic cell adhesion, small GTPase signaling all implicated in PanIN dysregulation. The transition from pancreatic ductal epithelial cells to PanIN is facilitated by telomere shortening and alterations in the KRAS signaling pathway. Mutational profiling of patient tissues reveals KRAS activation as an early event, present in over 99% of PanIN-I lesions. Progression from PanIN-I to PanIN-II is marked by the inactivating mutation in CDKN2A, while further progression to PanIN-III involves inactivating mutations in TP53 and SMAD4 crucial for TGF-β pathway activation. Once these accumulate, progression from PanIN to invasive PDAC ensues. While most tumors exhibit a predominant genetic alteration affecting a specific dysregulated signaling pathway, the specific variant of the mutated gene often varies among individual patients, contributing to the genetic heterogeneity observed in pancreatic cancer [4].

PDAC is classified into four stages extent of tumor spread, depicted in Figure 1. Stage I represents localized neoplasia confined to the pancreas, while Stage II involves regional spread to nearby lymph nodes. In Stage III, neoplasia infiltrates surrounding tissues and blood vessels, and in Stage IV, metastasis to distant organs occurs [3,4]. Advanced stage of PDAC is characterized by aggressive infiltration of surrounding tissues and blood vessels, leading to extensive invasiveness and metastasis to lymph nodes, liver, peritoneal cavity, lungs [2,19]. In contrast, pancreatic tumors originating from endocrine cells are uncommon, and typically exhibit excessive hormone secretion, most commonly insulin or glucagon [2,9,10].

### 3.2. Biosynthesis and Functions of miRNAs

**Figure 2:**
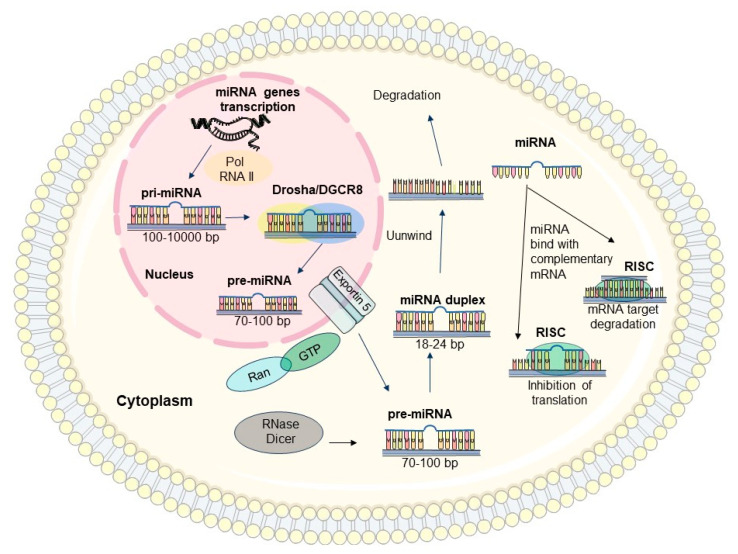
Synthesis of miRNA. The figure was partly generated using Servier Medical Art, provided by Servier, licensed under a Creative Commons Attribution 3.0 unported license.

miRNAs are encoded by endogenous sequences, usually located in intergenic regions and form separate transcription units with their own promoters and terminators. Some miRNA occurring within introns of other genes have also been identified. miRNAs synthetized from intronic RNA fragments during splicing have also been found. This type of intron processing involves RNase III-related enzymes, exonucleolytic cutting and probably RNA-mediated splicing. Intergenic miRNA is synthesized when the MIRNA gene is activated, the DNA strand opens up and the gene is transcribed by RNA polymerase II. The initial gene transcript is called primary miRNA (pri-miRNA) [21,22,23,24,25,26]. This is a strand containing 100–10,000 nucleotides and has at least one ‘hairpin’ structure. Next, the enzyme complex Drosha/DGCR8 cuts the nucleotides surrounding the ‘hairpin’ structure and this is how pre-miRNA is formed. It is composed of about 70–100 nucleotides. In the next step, the pre-miRNA is transported from the cell nucleus to the cytoplasm using exportin 5 and the Ran-GTP6 complex [21,22,23,24,25,26,27,28,29]. Once in the cytoplasm, the Dicer complex (type III RNase) catalyzes shortening of pre-miRNA to 18–24 nucleotides and the formation of a double-stranded miRNA (duplex), which contains the hairpin structure. The double-stranded miRNA interacts with the RNA-Induced Silencing Complex (RISC), which leads to their separation into two single miRNA strands, the first one is degraded and the second one binds directly with the target mRNA. In the mature miRNA molecule, there is a conservative, several-nucleotide (2–8 nucleotides) sequence usually complementary to the 3′-UTR segment of the mRNA [28,29,30,31,32,33]. Single microRNA can regulate several mRNAs and conversely one mRNA can be the target of multiple microRNAs. The attachment of a miRNA and RISC endonuclease complex to a target mRNA may have two consequences. In most cases, it activates the Dicer endonuclease in the RISC complex to hydrolyze the mRNA, which results in the post-transcriptional silencing of gene expression. After mRNA hydrolysis, the miRNA molecule is able to bind again to another mRNA molecule located in the cytoplasm. Less commonly, the binding of the RISC-miRNA complex to a target mRNA provides a spherical barrier of translation, which manifests as silencing of gene expression without mRNA degradation [28,34]. A schematic pattern of miRNA synthesis and function is depicted in Figure 2. More than 2500 different miRNAs have already been recognized in humans [24,25]. Due to the small size of miRNAs, they are transported actively into the extracellular space and into the blood in exosomes that prevents them from degradation. Consequently, miRNAprofiles can be analyzed in various biological fluids, cells, and tissues [28,33,34,35,36,37]. For instance, the serum of patients with non-small cell lung cancer exhibits the presence of 63 miRNAs that are absent in the serum of healthy people. In contrast, the serum of patients with colon cancer exhibits detectable levels of miRNA-29a, miRNA-221, miRNA-223, miRNA-25, miRNA-92 and miRNA-99a [38]. This finding is particularly relevant for cancer diagnostics. Moreover, the miRNA profile holds significant potential as a valuable diagnostic indicator in both acute and chronic pancreatitis [33,37,39,40,41].

### 3.3. Selected Pancreatic Cancer Pathways and Associated miRNAs

A comprehensive genomic analysis involving 24 pancreatic cancer patients revealed an average of 63 genetic alterations, per patient, predominantly comprising point mutations across various cellular pathways mutations in the Ras oncogene particularly oncogenic KRAS, represent the most prevalent oncogenic alterations observed in human cancers. Among the various signaling pathways activated by oncogenic KRAS, the phosphoinositide 3-kinase (PI3K) pathway emerges as a critical effector. The PI3K pathway, in turn, cross-activates other pathways, complicating the precise identification of downstream effectors specific to KRAS/PI3K activation. Signaling pathways often operate in a coordinated manner to orchestrate appropriate physiological responses to internal and external stimuli. This coordination involves cross-talk, where common proteins participate in multiple signaling pathways simultaneously. For instance, PTEN protein is implicated in regulating both AKT within the PI3/AKT pathway and the TGF-β receptor in the TGF-β pathway. Understanding these interactions between signaling pathways is crucial for elucidating the mechanisms underlying PDAC formation [42,43,44].

#### 3.3.1. PI3K/AKT Signaling Pathway

This pathway is stimulated by external factors such as insulin, cytokines, growth factors including PDGF (platelet-derived growth factor), EGF (epidermal growth factor), bFGF (basic fibroblast growth factor), VEGF (vascular endothelial growth factor), IGF-1 (insulin-like growth factor 1), which bind to their respective membrane kinase receptors on the various cells. The AKT pathway is also important in diabetes, as AKT affects cellular metabolism [44]. AKT (known as PKB–Protein Kinase B) is a serine/threonine kinase responsible for protein phosphorylation. PI3K (phosphatidylinositol 3-kinase) is involved in the phosphorylation of PIP2 (phosphatidylinositol 4,5-bisphosphate) to PIP3 (phosphatidylinositol 3,4,5-triphosphate) with the ATP (Adenosine triphosphate) as a phosphate group donor. PIP3 directly recruits AKT to the cell membrane and interacts with PDK1 (Phosphoinositide-dependent kinase 1), which is the main activator of AKT. Active AKT is a proto-oncoprotein with many substrates and effects, for example: inhibition of Bax which prevents from apoptosis, phosphorylation of FOXO (Forkhead box protein O1), a protein acting as a tumor suppressor that is responsible for inhibition of proliferation. Phosphorylated FOXO becomes ubiquitinated and is degraded in the proteasome. The active PI3K/AKT pathway stimulates mTOR kinase (mechanistic target of Rapamycin) which activates an increase of HIF1 (hypoxia-inducible factor 1), leading to the overexpression of GLUT1 (glucose transporter 1). This enables cancer cells to uptake more glucose, providing an energy source for growth.

The PI3K-AKT-mTOR pathway is negatively regulated by the product of the tumor suppressor gene *PTEN* (phosphatase and tensin homolog), which dephosphorylates PIP3 to PIP2, thereby inhibiting PDK1 and subsequently AKT, resulting in reduced cell proliferation [44,45,46,47].

**Figure 3:**
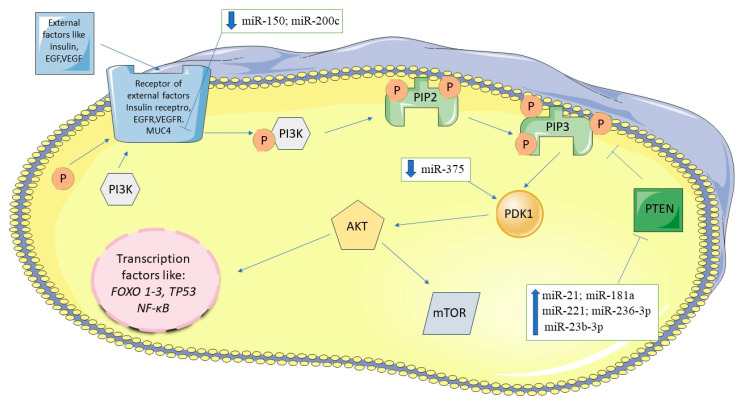
PI3K/AKT signaling pathway and miRNAs in pancreatic cancer. The figure was partly generated using Servier Medical Art, provided by Servier, licensed under a Creative Commons Attribution 3.0 unported license.

*PTEN*, a crucial tumor suppressor gene, is targeted directly by several microRNAs including miRNA-21, miRNA-181a and miRNA-221. Studies conducted on PDAC tissues have consistently reported elevated levels of miRNA-21 and miRNA-221 [48,49,50]. Increased expression of miRNA-21 has been demonstrated to downregulate *PTEN* and *PDCD4* (programmed cell death 4), promoting invasion and metastasis in PDAC [51]. In 2020, Zhang et al. identified that miRNA-23b-3p downregulated PTEN expression, thereby affecting the PI3K/AKT pathway in PANC-1 cells [52]. Similarly, inhibition of miRNA-221 in PANC-1 and MiaPaCa-2 cell lines resulted in increased expression of tumor suppressors such as p27, p57, PUMA (the p53 upregulated modulator of apoptosis) and PTEN subsequently inhibiting cell proliferation. Studies performed on pancreatic cell lines, PANC-1 and MiaPaCa-2, showed that silencing of miRNA-221 resulted in inhibition of proliferation of these cells [48,49]. Further investigations on pancreatic cell lines PANC-1 and MiaPaCa-2 revealed that miRNA-181a promotes tumor migration and metastasis, whereas miRNA-200c and miRNA-375 inhibit tumor proliferation [50,53,54,55]. Specifically, miRNA-375 targets directly *PDK*1 and miRNA-200c targets *MUC4* (Mucin 4), influencing epithelial–mesenchymal transition and metastatic potential in pancreatic cancer cells [53,54]. miRNA-200c directly targets *MUC4* causing activation of AKT. This causes an increase in the expression of N-cadherin, an adhesion factor that enables cancer cells to metastasize to distant organs [14,53,54,55,56,57]. The downregulation of miRNA-150 was found to correlate with increased levels of MUC4, an oncoprotein inhibiting pancreatic cancer cell growth [58]. In PANC-1 cells overexpression of miRNA-873 was shown to silence *PLEK2* (Pleckstrin 2) gene expression, which interacts with AKT thereby inhibiting the PI3K/AKT pathway and reducing cell proliferation and promoting apoptosis in PANC-1 cell line [59]. Additionally, studies encompassing various pancreatic cancer cell lines (CFPAC-1, BxPC-3, PANC-1, SW1990) and 115 patient tissues demonstrated that decreased expression of miRNA-382 correlates with increased levels of *ANXA3* (Annexin A3), an activator of the PI3K/AKT pathway. Conversely, upregulation of miRNA-382 and inhibition of *ANXA3* led to PI3K/AKT pathway inhibition [60]. Figure 3 summarizes the interplay between the PI3K/AKT signaling pathway and associated miRNAs in pancreatic cancer.

#### 3.3.2. KRAS Signaling Pathway

KRAS (Kirsten rat sarcoma virus) pathway plays a pivotal role in the proliferation, invasion and metastasis of pancreatic cancer cells. The Ras protein, encoded by *KRAS*, is a small GTPase (21 kDa) attached to the cell membrane. Activation of the KRAS signaling pathway, typically initiated by growth factors such as the epidermal growth factor receptor (EGFR), involves guanine nucleotide exchange factors (GEFs) like GRB2 (growth factor receptor bound protein 2) and SOS (son of sevenless). These factors facilitate the exchange of GDP (guanosine diphosphate) for GTP on inactive Ras, thereby activating it. Once activated, RAS recruits and activates B-RAF protein, triggering a cascade of signaling events that regulate signaling pathways such as PI3K-AKT. Physiologically the activity of Ras is transiently regulated by GTPase activating proteins (GAPs), which promote the hydrolysis of GTP to GDP, thereby inactivating Ras shortly after activation [61,62,63,64,65]. Dysregulation of the KRAS pathway often involves insensitivity of Ras to GAP-mediated inactivation, resulting in constitutive activation of KRAS signaling [64,65]. Mutations in the KRAS gene are prevalent in approximately 90% of PDAC cases, irrespective of tumor stage [65].

**Figure 4:**
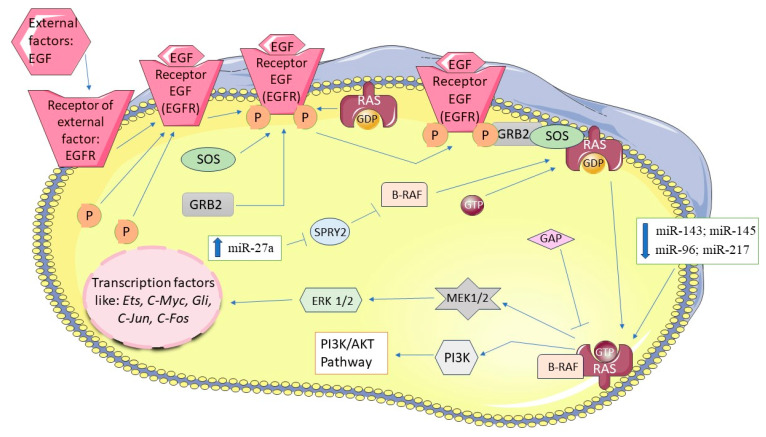
KRAS signaling pathway and miRNAs in pancreatic cancer. The figure was partly generated using Servier Medical Art, provided by Servier, licensed under a Creative Commons Attribution 3.0 unported license.

Several studies have highlighted the regulatory roles of miRNAs including miRNA-27a, miRNA-96, miRNA-126, miRNA-143, miRNA-145, miRNA-193b, miRNA-206, miRNA-217, miRNA-3923, Let-7a and Let-7b in the KRAS signaling pathway [66]. For instance, Ma Y. et al. demonstrated elevated expression of miRNA-27a in pancreatic cancer cells, where it targets *SPRY2* (Sprouty2), a negative regulator of the KRAS pathway. In cell lines such as PANC-1, MIA PaCa-2, and HEK293T, downregulation of miRNA-27a inhibited cell growth and migration [67]. Furthermore, miRNA-217 has been shown to correlate directly with *KRAS* expression levels in PANC-1, MIA PaCa-2, AsPC-1 and BxPC-3 cells. Reduced expression of miRNA-217 was observed in PDAC tissues compared to normal pancreatic cells. Upregulation of miRNA-217 led to decreased *KRAS* expression and subsequently reduced AKT phosphorylation, thereby inhibiting cell proliferation and promoting cell survival in these cell lines [68]. Studies have also reported interactions between miRNA-96, miRNA-126, Let-7 family members with the 3′-UTR region of *KRAS* [69,70,71,72,73]. miRNA-96 expression is notably low in PDAC tissues, and restoration of its expression inhibited proliferation of PANC-1 and CFPAC-1 cells [69,70]. Similarly, reduced expression of miRNA-206 has been observed in PDAC compared to normal pancreatic tissue. Overexpression of miRNA-206 suppressed cell cycle progression at the G0/G1 phase and downregulated KRAS expression in PANC-1 and PANC10.05 cells [74]. The interactions between miRNAs and KRAS pathway components are illustrated in Figure 4.

#### 3.3.3. JAK/STAT Signaling Pathway

The Janus kinase/signal transducers and activation transcription (JAK/STAT) controls cellular processes such as cell differentiation, tissue regeneration, inflammation, proliferation and apoptosis. The activation and subsequent cellular outcomes of this pathway depends are contingent upon the specific isoforms of JAK or STAT proteins that are engaged. In neoplastic conditions, this signaling pathway is frequently overactivated. The JAK/STAT pathway can be activated by over 50 distinct biomolecules, interferons (e.g., IFN-γ, IFN-α/β), interleukins (e.g., IL-2, IL-3, IL-4, IL-5, IL-6, IL-7, IL-9, IL-17), and various growth factors (e.g., erythropoietin (EPO), growth hormone (GH), prolactin and thrombopoietin (TPO)). Upon the binding of these signaling molecules to their respective membrane cytokine receptors, JAK proteins are recruited to the receptor surface. Once associated with the receptor, JAK proteins undergo phosphorylation. This leads to the phosphorylation of the tyrosine residues on the cytokine receptor via the JAK proteins. Subsequently, the JAK proteins themselves undergo a secondary phosphorylation. This phosphorylation cascade facilitates the recruitment of STAT proteins to the cytokine receptor, where they are then phosphorylated by JAK kinases. This phosphorylation cascade facilitates the recruitment of STAT proteins to the cytokine receptor, where they are then phosphorylated by JAK kinases. Phosphorylated STAT proteins, through their SH2 domains, form dimers and dissociate from the cytokine receptor. These phosphorylated STAT dimers translocate to the nucleus and modulate the transcription of target genes such as *NOS2*, *MYC*, *SOCS* and *p21*. In tumorigenesis, this pathway is often dysregulated, and STAT phosphorylation can occur through alternate receptors such as EGFR or SRC [75,76,77]. Negative regulation of the JAK/STAT pathway is mediated by the SOCS (suppressor of cytokine signaling) family of proteins, which includes eight members: SOCS1-SOCS7 and CIS. These proteins contain a central Srchomology 2 (SH2) domain and a C-terminal SOCS box. The expression of SOCS proteins, particularly CIS, SOCS1, SOCS2 and SOCS3, is induced in response to various cytokines. Overexpression of these proteins serves to inhibit JAK/STAT signaling, thereby establishing a negative feedback loop to prevent hyperactivation of the pathway [75,78].

**Figure 5:**
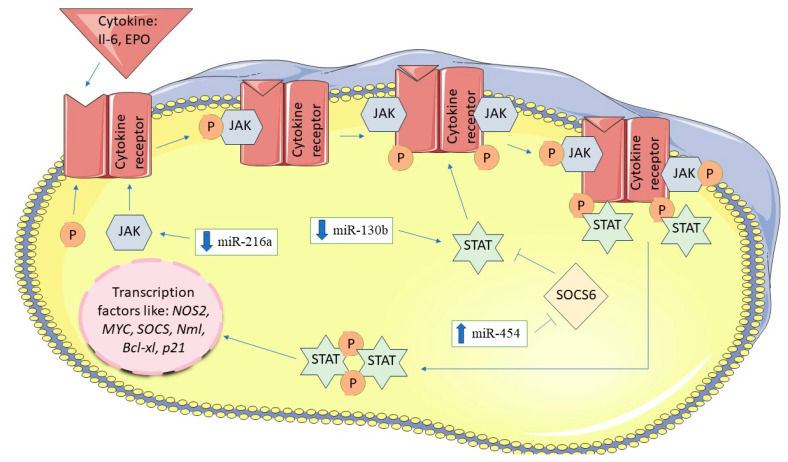
JAK/STAT signaling pathway and miRNAs in pancreatic cancer. The figure was partly generated using Servier Medical Art, provided by Servier, licensed under a Creative Commons Attribution 3.0 unported license.

Several miRNAs have been identified as a target of the JAK/STAT signaling pathway. miRNA-216a, which directly targets *JAK2* has been found to be downregulated in PDAC. The upregulation of miRNA-216a inhibits transcriptional genes controlled by the JAK/STAT pathway, including survivin, which is responsible for suppression of apoptosis [79]. miRNA-130b, which binds directly to the 3′-UTR site of the *STAT3*, was discovered to be downregulated in PDAC tissue and cell lines PANC-1 and ASPC-1. The downregulation of this miRNA was associated with poor prognosis and tumor progression [75,79,80]. Restoration of miRNA-130b expression leads to the inhibition of cell proliferation [80]. Another miRNA, miRNA-155, belonging to the oncogenic miRNA family, was demonstrated by Huang et al. to cause decreased expression of *SOCS1* and activation *STAT3* thereby increasing the metastatic potential of tumors [81]. Studies conducted on PANC-1 and AsPC-1 cell lines showed that upregulation of miRNA-1225, which targets the 3′-UTR region of the *JAK1* gene reduces their proliferation and induces apoptosis [82]. It was also shown that miRNA-203 was upregulated in PANC-1 and BXPC3 cell lines with a concomitant downregulation of *SOCS3* expression [78]. Studies on PANC1, Mia-PaCa-2, AsPC-1, BXPC-3, SW1990 lines suggest that *SOCS5* is a target of miRNA-301a [83]. According to the result of the study conducted by Wu et al. miRNA-424-5p is an oncogenic molecule, whose overexpression in pancreatic cancer has been reported to downregulate the SOCS6 protein what further increased ERK signaling pathway activity [84]. JAK/STAT signaling pathway and related miRNAs are presented in Figure 5.

#### 3.3.4. TGF-β Signaling Pathway

The TGF-β signaling pathway plays a crucial role in cell growth, apoptosis and differentiation particularly during embryonic development. This pathway is activated by proteins from the TGF-β family, including TGF-β1/2/3, activins, NODAL homolog, bone morphogenic proteins (BMPs), growth and differentiating factors (GDFs) and anti-Mullerian hormone. These proteins are synthesized as preproteins that, following proteolytic processing, form a heterotetramer composed of two proproteins. This complex binds to the TGF-beta binding protein (LTBP) which, under the influence of an extracellular signal, facilitates the formation of the mature TGF-β family protein dimer. These changes occur in the intercellular space. The TGF-β ligand binds to its cell membrane receptor TβR, which contains two domains: TβRI and TβRII. Subsequently, TβRI phosphorylates TβRII, recruiting cytoplasmic protein from to SMADs (Suppressor of Mothers Against Decapentaplegic) family, which are then phosphorylated. In humans, there are eight isoforms of the SMAD protein known as R-SMADs. Phosphorylated SMAD2 and SMAD3 form a complex with SMAD4 (Co-SMAD), and this complex then translocates to the cell nucleus, where it binds to DNA and either activates or inhibits gene transcription. For instance, this pathway negatively regulates the expression of C-Myc and promotes the expression of cell cycle inhibitors such as p15 and p21 [85,86,87,88,89,90,91,92]. Approximately 55% of pancreatic cancers have a mutation in Co-SMAD, resulting in the reduced formation or complete absence of the SMADs protein complex in the cytoplasm [93].

**Figure 6:**
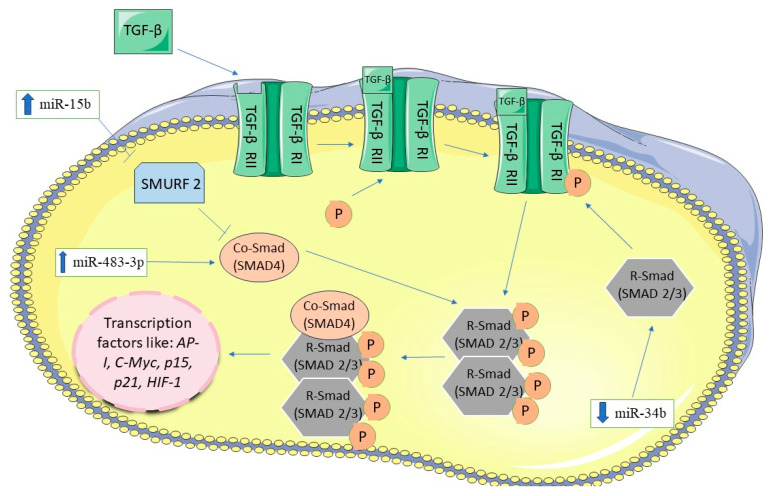
TGF-β signaling pathway and miRNAs in pancreatic cancer. The figure was partly generated using Servier Medical Art, provided by Servier, licensed under a Creative Commons Attribution 3.0 unported license.

The activation of the TGF-β pathway has been shown to downregulate miRNA-29c, leading to a constitutively activated Wnt/β-Catenin pathway which promotes growth of the PANC-1 cell line. miRNA-29c targets *LRP6*, *FZD4*, *FZD5*, *R-SMAD*, suggesting a cross-talk between TGF-β and Wnt/β-catenin signaling pathway [94]. Activation of the TGF-β pathway has been associated with EMT (epithelial–mesenchymal transition) induction, through the stimulation of SMADs proteins [91,92]. In 2015, Zhang et al. revealed that *SMURF2* (SMAD Specific E3 Ubiquitin Protein Ligase 2) *is* a target of the miRNA-15b, SMURF2 functions as an inhibitor of SMADs [95]. Furthermore, the activated TGF-β pathway has been linked to the overexpression of miRNA-155, which induced tumor invasion and metastasis in NMuMG cell line [96]. In vitro studies performed on the NIH-3T3 cell line have demonstrated that *SMAD4* is a direct target of miRNA-199a [97]. Hao et al. found that miRNA-483-3p inhibits *SMAD4* expression during tumor progression in NIH-3T3 cell line [98]. Interestingly, the maturation of miRNA-21 involving the DROSHA complex and RNA helicase p68 is induced via the TGF-β signaling pathway. This induction leads to increased miRNA-21 expression what further downregulated suppressor genes such as *PTEN* and *PDCD4* (programmed cell death 4), promoting invasion and metastasis in PASMC cell line [51]. Decreased miRNA-494 expression has been detected in PDAC tissue, and this reduction leads to increased *FOXM1* (Forkhead Box M1), which subsequently increases β-Catenin expression [99]. Moreover, TGF-β induced upregulation of miRNA-216a has been observed in AR42J cells and in a mouse model of pancreatic cancer. *PTEN* and *SMAD7* have been identified as potential targets of miRNA-216a. Transfection of AR42J cells with miRNA-216a mimetics downregulated *PTEN* and *SMAD7* expression, resulting in increased expression of AKT protein and TGF-βI receptor. Conversely, inhibition of miRNA-216a leads to upregulation of *PTEN* and *SMAD7* expression [100]. These findings emphasize the cross-talk between TGF-β and PI3K/AKT pathways. Additionally, miRNA-23a-3p has been found to promote tumor cell proliferation, invasion and migration. Elevated levels of miRNA-23a have been detected in PANC-1, MIA Paca-2, BxPC-3, SW1990 lines and PDAC patient tissues. This upregulation of miRNA-23a correlates with downregulation of TGF-βRII expression at the protein level [101]. The TGF-β signaling pathway and associated miRNA are illustrated in Figure 6.

#### 3.3.5. Wnt/β-Catenin Signaling Pathway

This pathway is essential for proliferation and differentiation during embryonic development. In cancer cells, it plays a pivotal role in proliferation, invasion and metastasis. Under physiological conditions, the activation of this pathway begins with the binding of the extracellular factor Wnt to a receptor complex composed of the Frizzled receptor (FZD), low-density lipoprotein receptor-related proteins 5 and 6 (LPR 5/6) and the Dishevelled segment polarity protein 1 (DVL) protein. The activation of DVL protein results in the dissociation of the Axin-GSK3β-β-Catenin-APC-CK1α complex (GSK3β-glycogen synthase kinase-3β; APC-adenomatous polyposis coli; CK1α-casein kinase 1 α). This dissociation releases active β-Catenin into the cytoplasm. Subsequently, β-Catenin translocates to the cell nucleus, where it influences transcriptional genes including, MYC and cyclin D1. In tumors, the activation of the pathway is aberrant due to mutations in genes encoding components of Axin-GSK3β-β-Catenin-APC-CK1α complex. For example, mutation e.g., in APC results in the dissociation of β-Catenin from Axin-GSK3β-β-Catenin-APC-CK1α complex without stimulation by Wnt factor, thereby leading to constitutive activation Wnt/β-Catenin signaling pathway [102,103,104,105].

**Figure 7:**
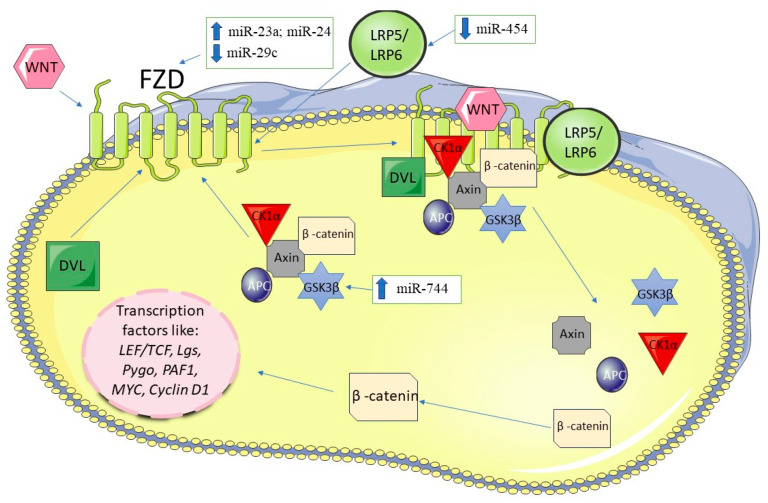
Wnt/β-Catenin signaling pathway and miRNAs in pancreatic cancer. The figure was partly generated using Servier Medical Art, provided by Servier, licensed under a Creative Commons Attribution 3.0 unported license.

Studies using Wnt3a, an activator of Wnt/β-catenin signaling, have demonstrated that miRNA-29a induced resistance to gemcitabine in MIAPaCa-2, PSN-1, BxPC-3 and PANC-1 cell lines [106]. Overexpression of miR-15a down-regulates Wnt3a, leading to reduced cellular proliferation and survival through the Wnt/β-catenin signaling pathway. [107]. Downregulation of miRNA-27a has been observed to inhibit the Wnt/β-catenin pathway, promoting proliferation and suppressing apoptosis in PANC-1 cells through the Wnt/β-catenin pathway by target: cyclin D1, Bax, Wnt, β-catenin proteins [108]. Several studies have indicated that miRNA-29c targets FRATL (regulator of Wnt signaling pathway 1 ligand), LRP-6, FZD4 and FZD5, crucial components that activate the Wnt/β-Catenin signaling pathway [94,109], suggesting a functional interplay (cross-talk) between the TGF-β and Wnt/β-Catenin signaling pathways. Furthermore, a microarray analyses have shown that miRNA-23a and miRNA-24 regulated the expression of *FZD5* directly or indirectly [110]. Conversely, decreased level of miRNA-195 have been associated with increased expression of C-myc, a downstream target of Wnt signaling in in PANC-1 and AsPC-1 cells [111] Reduced level of miRNA-148a has also been linked to inhibition of Wnt signaling in pancreatic cancer, with its restoration leading to decreased tumor growth and invasion by downregulating β-catenin and C-myc levels in PANC-1 cells [112]. In 2017, Fan et al. identified that miRNA-454 downregulates *LRP6* in PANC-1 cell line [113], while miRNA-940 has been shown to downregulate *GSK3β* in SW1990 cells, thereby promoting cell growth [114]. Moreover, Zhou et al.’s study across various pancreatic cancer cell lines highlighted miRNA-744 as a negative prognostic factor, demonstrating its activation of the Wnt/β-catenin pathway by targeting *SFRP1* (secreted Frizzled related protein 1) and *GSK3β* [109]. Figure 7 illustrates the Wnt/β-catenin signaling pathway and the miRNAs associated with its regulation.

### 3.4. Role of miRNA in Cellular Processes Associated with Carcinogenesis

During tumorigenesis, a pivotal event is the acquisition of capabilities by a cell to evade control mechanisms governing cell cycle progression, leading to disruptions in proliferation and apoptosis. These abnormalities stem from genetic mutations that either deactivate suppressor genes, activate proto-oncogenes, cause inhibition gene transcription or induce constitutive gene activity. Consequently, cells harboring these mutations exhibit altered genotypes and phenotypes compared to normal cells, thereby transforming into cancerous cells. These cancer cells typically demonstrate insensitivity to growth inhibitory signals, evade programmed cell death (apoptosis), exhibit metastatic potential and possess angiogenic capabilities. Therefore, the segment of this study aims to elucidate the role of miRNAs in fundamental cellular processes intricately linked with tumorigenesis.

#### 3.4.1. Cell Cycle

**Figure 8:**
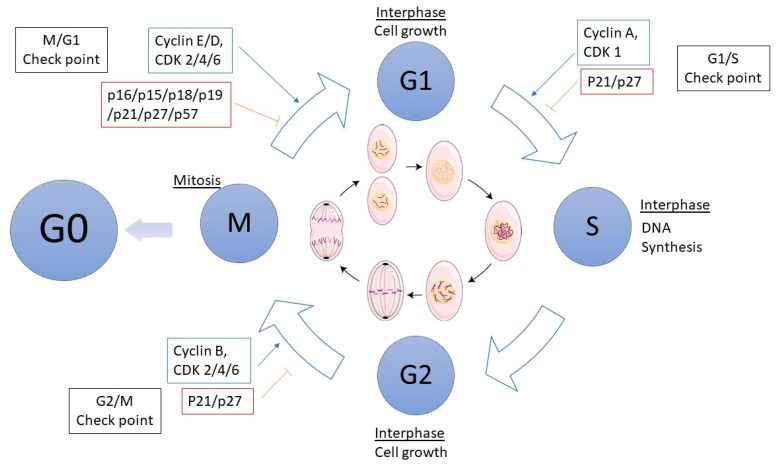
The scheme of cell cycle phases. Interphase: M/G1 checkpoint = initiation of the cell cycle; G1 phase = cell growth and protein synthesis; G0 phase = differentiation, rest from divisions; G1/S checkpoint = repair of errors in DNA; S phase = DNA replication; G2 phase = tubulin synthesis; G2/M checkpoint = repair of errors after replication. M phase (Mitosis): karyokinesis, cytokinesis, division of a cell into two cells. The figure was partly generated using Servier Medical Art, provided by Servier, licensed under a Creative Commons Attribution 3.0 unported license.

The regulation of cell cycle progression in normal cells is delicately maintained by the interplay between suppressor genes, known as anti-oncogenes and protooncogenes, which respectively inhibit or promote cell growth and differentiation. The cell cycle comprises distinct phases: interphase (including G1, S, G2 phases) and mitosis (M phase) (consisting of prophase, prometaphase, metaphase, anaphase, telophase and cytokinesis), with cells exiting the cycle into a resting state known as G0 (quiescent cell) upon completion of proliferation. Throughout proliferation, cells navigate checkpoints overseen by two primary groups of proteins: cyclins and cyclin-dependent kinases (CDKs), which are critical regulators of cell cycle activation. M (or G0)/G1 checkpoint regulates the entry of a G0 cell back into the cell cycle. Cells in G0 are in a state of dormancy and are not actively preparing to divide. When conditions are favorable, cells receive the necessary signals to re-enter the cell cycle at the G1 phase. During the G1 phase, the cell grows and synthesizes essential organelles, mRNAs and proteins required for subsequent phases. At the G1/S checkpoint, stringent monitoring sensures the presence of necessary proteins and verifies the integrity of DNA. Upon detection of abnormalities,, mechanisms are initiated; failure to repair damaged DNA triggers apoptosis, thereby preventing the proliferation of cells carrying damaged genetic material cells that pass through the G1/S checkpoint with unrepaired DNA damage pose a significant risk of becoming cancerous. In the S phase, DNA replication occurs, resulting in the duplication of genomic DNA into two identical copies. Subsequently, cells progress to the G2 phase, during which they prepare for division by synthesizing tubulin, a protein essential for forming microtubules. Microtubules organize into spindle fibers during mitosis (M phase), facilitating the segregation of chromosomes to opposite poles of the cell. The transition from G2 to M phase is regulated by the G2/M checkpoint, which ensures the fidelity of newly synthesized DNA molecules. During the M phase, the cell undergoes division, characterized by the separation of the nucleus (karyokinesis) and subsequently the cytoplasm (cytokinesis) to form two distinct daughter cells. Karyokinesis involves the formation of new nuclear membranes around the segregated sets of chromosomes, derived from the remnants of the original nuclear membrane. Concurrently, cytokinesis involves the formation of a contractile ring that leads to the division of the cell membrane and the physical separation of the two daughter cells. Following M phase, cells may re-enter the cell cycle (S phase), enter a quiescent state (G0) or undergo differentiation. A critical regulator at several cell cycle checkpoints is the p53 protein, which plays a pivotal role in preventing cells with damaged DNA from progressing to the next phase. Upon detecting DNA damage, p53 halts the cell cycle at the checkpoint by inducing the production of cyclin-dependent kinase inhibitor (CKI) proteins. CKIs bind to CDK-cyclin complexes, inhibiting their activity and allowing time for DNA repair. Additionally, p53 activates DNA repair enzymes to facilitate the restoration of damaged DNA. If DNA repair proves impossible, p53 triggers apoptosis to prevent the propagation of cells carrying genetic defects [19,58,115,116,117]. Figure 8 shows a schematic representation of the cell cycle and its checkpoints.

miRNAs are critical regulators of the cell cycle and cellular proliferation. Studies utilizing the HS766T cell line have demonstrated that miRNA-21 negatively regulates the *PTEN* tumor suppressor gene [48]. Similarly, miRNA-486 has been shown to control cell proliferation by targeting *PTEN* in the Capan-2 cell line (human pancreatic ductal adenocarcinoma cells) [118]. According to research conducted by Halkova et al., miRNA-424-5p caused downregulation of SOCS6 protein, leading to increased activation of the ERK pathway, which plays an important role in cell proliferation and migration [119]. The upregulation of miRNA-27a has been detected to inhibit the expression of the suppressor gene *SPRY2* in PDAC tissue, as well as in the PANC-1 and SW1990 cell lines [84]. Studies performed by Basu et al. and Su et al. have demonstrated that elevated expression of miRNA-221 inhibits the translation of CDK N1B, an inhibitor of CDK1, thereby promoting the transition from G1 to S phase in BxPC-3 and CFPAC-1 cell lines [120,121]. Another miRNA involved in cell cycle progression is miRNA-203. A decreased level of miRNA-203 has been reported to activate cell progression to the G1 phase, while its downregulation inhibits the proliferation of CFPAC-1 cells [119]. Cyclin E2, a protein involved in the G1/S phase of the cell cycle is directly regulated by miRNA-26a and indirectly through ubiquitination by FBXW7 (F-Box and WD repeat domain containing 7). *FBXW7* is a crucial tumor suppressor and one of the most deregulated ubiquitin-proteasome system proteins in human cancer and is regulated by miRNA-223 [43,122,123]. Additionally, cyclin E2 expression is influenced by miRNA-21, which regulates p27 expression, and miRNA-222, which regulates p57 expression. The upregulation of cyclin E2 leads to uncontrolled proliferation [48,124,125,126]. According to a study conducted by Liffers et al., miRNA-148a was downregulated in IMIM-PC2 cells and targets the 3-UTR of *CDC25B* (cell division cycle 25B). CDC25B is an activator of CDK1 (cyclin-dependent kinase 1), which stimulates the transition from G1 to the S phase, resulting in the activation of proliferation [127]. miRNA-143, miRNA-126 and let-7-d have been shown to target elements of the KRAS pathway and are involved in the control of cell proliferation [71,119,128]. In 2009, Lee et al. identified that miRNA-107 was downregulated in PDAC tissue targeting *CDK6*, which encodes a cyclin D1-dependent kinase, leading to the stimulation of growth of MiaPACA-2 and PANC-1 cells [129]. Other miRNAs detected in PDAC patients tissue involved in the cell cycle control are miRNA-132 and miRNA-212, which were found to be upregulated; however, their inhibition in PANC-1 cell lines resulted in reduction of tumor cell proliferation [130]. Additional in vitro studies involving AsPC-1, BxPC-3, PANC-1, MiaPaCa-2 and SW1990 lines have shown that overexpression of miRNA-141 led to the inhibition of the cell cycle at the G1 phase and activation of apoptosis through the reduction of Bcl-2 and promotion of cyclin D1, [131]. The involvement of miRNA in the cell cycle, as investigated in PDAC cell lines and samples from patients, is summarized in Table 1.

#### 3.4.2. Programmed Cell Death

**Figure 9:**
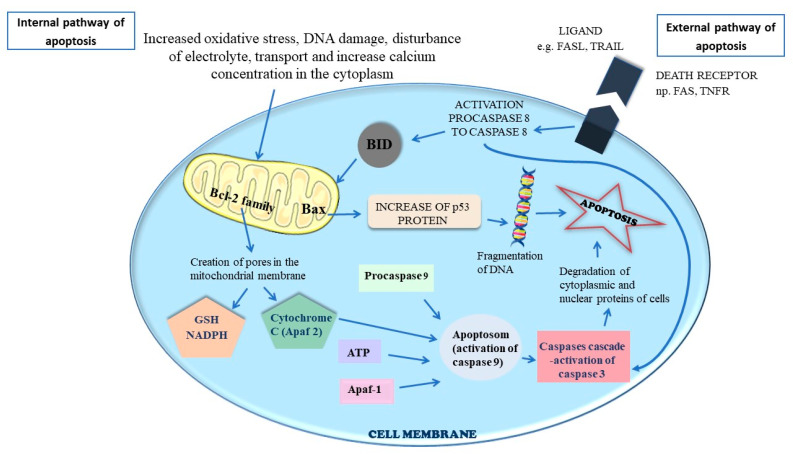
The internal and external apoptosis pathways.

Apoptosis is a programmed cell death mechanism activated in response to irreversible cellular damage, ensuring the removal of dysfunctional cells that can no longer perform their physiological functions. Apoptosis comprises three stages: initiation, execution and degradation. It can be triggered by either internal (mitochondrial) or external factors. The internal pathway is typically initiated by irreversible DNA damage often caused by excessive oxidative stress, mistakes during proliferation. Increased oxidative stress, DNA damage, disturbance of electrolyte transport and increase calcium concentration in the cytoplasm, lead to the recruitment of ATM (the ataxia telangiectasia-mutated protein kinase) and p53 proteins to address the damaged DNA. miRNAs interact indirectly with p53 protein expression via a family of growth inhibitors (INGs), which are also tumor suppressor proteins that induce cell growth arrest and/or apoptosis [135]. p53 promotes the proteolytic cleavage of proapoptotic proteins e.g., Bax (bcl-2-like protein 4), Bid (BH3 interacting-domain death agonist), Bad (BCL2 associated agonist of cell death), resulting in the formation of pores in the mitochondrial membrane. This leads to the uncontrolled release of all ions (e.g., Ca^2+^) and proteins (including cytochrome C) into the cytoplasm. Released cytochrome C recruits APAF (apoptotic protease activating factor-1) and procaspase 9. Caspases are a family of cysteine proteases that function in a cascade triggered by apoptosis signaling. The culmination of this cascade is the cleavage of a numerous proteins in the cell, followed by cell disassembly, death and the subsequent phagocytosis and removal of the cell debris. Caspase 9 not only causes protein destruction, but also activates other procaspases, leading to their proteolytic cleavage into active caspases, thus perpetuating the caspase cascade. This cascade results in the degradation of many cellular proteins in the cell, leading to cell shrinkage, chromatin condensation and the formation of apoptotic bodies, which are phagocytosed by macrophages. The process of apoptosis is inhibited by survivin, a member of the inhibitor of apoptosis (IAP) family [136,137,138,139,140,141,142]. The internal and external pathway of apoptosis is illustrated in Figure 9.

The external apoptosis pathway is initiated by death receptor-dependent pathways, such as FASL (CD95 ligand) binds with FASR (apoptosis antigen 1), TRAIL (TNF-related apoptosis-inducing ligand) interlocks with the death receptors DR4 (TRAIL-RI) or DR5 (TRAIL-RII) and TNF (tumor necrosis factor) conjoins with TNFR (tumor necrosis factor receptor). Upon activation death receptor recruits death factors such as TRADD (tumor necrosis factor receptor type 1-associated DEATH domain protein), FADD (FAS-associated death domain protein) and procaspase 8 to form the death-inducing signaling complex (DISC). This leads to proteolytic cleavage of procaspase 8 to caspase 8, which causes a caspase cascade. Both caspase 8 and caspase 9 initiates procaspase 3 to caspase 3. Caspase 3 then activates DNase, which fragments DNA into equal pieces of approximately 180 base pairs, and also degrades the cytoskeletal proteins. Ultimately, apoptotic bodies containing fragments of proteins, DNA, mitochondria and other intracellular elements are formed and subsequently phagocytosed by macrophages and other phagocytes [136,137,138,139,140,141,142].

Numerous studies on PDAC have focused on the identifying of miRNAs associated with apoptosis process. The expression of miRNA-34a has been reported to be significantly downregulated in PANC-1 and SW-1990 cells correlating with decreased expression of Notch1 (neurogenic locus notch homolog protein 1) and Notch2 (neurogenic locus notch homolog protein 2) proteins. These proteins, upon activation, are transported to the nucleus, where they bind to transcription factors and regulate the expression of genes involved in cell growth, apoptosis and differentiation [143,144]. Another miRNA, miRNA-23a has been found to regulate APAF1, a protein responsible for the activation of procaspase 9 to caspase 9 during apoptosis [145]. miRNA also influence the expression of members of the Bcl-2 family proteins that regulate apoptosis. The antiapoptotic members include Bcl-2 (B-cell lymphoma 2) and Bcl-xL (B-cell lymphoma-extra large), while pro-apoptotic members include Bax (bcl-2-like protein 4), BAK (Bcl-2 homologous antagonist/killer)), Bim (Bcl-2-like protein 11), Bid (BH3 interacting-domain death agonist) and BAD (BCL2 associated agonist of cell death). Members of the Bcl-2 family share one or more of four characteristic domains of homology called the Bcl-2 homology (BH) domains (designated BH1, BH2, BH3 and BH4). The anti-apoptotic Bcl-2 and Bcl-xL proteins, contain all four BH domains. The BH domains also serve to subdivide the pro-apoptotic Bcl-2 proteins into those with multiple BH domains such as Bax and BAK or those with only the BH3 domain such as Bim, Bid and BAD. In PDAC, *BIM* expression is significantly downregulated by miRNA-24. The decreased level of miRNA-24 results in increased expression of BIM promoting apoptosis in PANC-1 cells [146]. Several studies have reported that miRNA-21 leads to the overexpression of Bcl-2, while miRNA-181b caused inhibition of Bcl-2 in MIA PaCa-2 [147,148,149]. Elevated levels of miRNA-301a have been detected in tissues from pancreatic cancer patients. In turn, studies conducted on the BxPC-3 cell line (exhibiting epithelial morphology that was isolated from the female patient with adenocarcinoma) indicated that miRNA-301a directly binds to the 3′-UTR fragment of *BIM* reducing its expression and promoting cell proliferation. Conversely, the downregulation of miRNA-301a activates apoptosis [150]. The role of miRNAs in the apoptosis in pancreatic cancer is summarized in in Table 2.

#### 3.4.3. Migration and Metastasis

Cancer cells require increased space for growth due to their ability to proliferate uncontrollably. Each cell division elevates the likelihood of mutations within the cancer cell genome, leading to further alterations in gene expression. Initially, cancer cells proliferate locally to form a tumor. As the tumor grows, it invades the basement membrane. Subsequently, cancer cells detach from their original site and migrate to lymphatic nodes or circulate through the bloodstream to nearby and distant organs, forming new tumor aggregates called metastases. This process occurs as a result of epithelial-mesenchymal transition (EMT), wherein pancreatic epithelial cells acquire mesenchymal cell characteristics, resulting in the loss of E-cadherin expression. The reduction in cell adhesion facilitates the migration of cancer cells to distant organs.

The overexpression of miRNA-10a has been observed to down-regulate the transcriptional suppressors and metastatic factors (homeobox transcription factors)—HOX (HOXB1, HOXB2, HOXA1) in various pancreatic cancer cell lines including, PANC-1, CAPAN-1, SUIT-2, KP-2 and MIA PaCa-2. HOX proteins play critical roles in regulating transcription factors involved in embryonic development, proliferation and apoptosis [156,157]. Previous studies have highlighted that the transmembrane protein MUC1 regulates the expression of at least 103 different miRNAs including miRNA-141 and miRNA-200c. Reduced levels of miRNA-141 and miRNA-200c have been significantly reduced in PDAC patients tissue presenting distant metastases. This reduction correlates with increased expression of *ZEB1* (zinc finger E-box binding homeobox 1), a key inducer of EMT that down-regulates E-cadherin in pancreatic cancer cells [95,158]. In vitro investigations have reported that metastatic cells overexpress of miRNA-194, miRNA-429, miRNA-200b, miRNA-200c [159]. Studies on PDAC tumor tissues have demonstrated that metastatic tissues present a downregulation of miRNA-34b, which promotes metastasis formation by upregulating SMAD 3 translation [159]. Additionally, decreased expression of miRNA-143 in metastatic pancreatic cancer tissues leads to activation of the KRAS signaling pathway and elevation of E-cadherin levels [128]. Research has shown that downregulation of miRNA-126 induces EMT by targeting *ADAM9* (disintegrin and metalloproteinase domain-containing protein 9) a protein involved in intercellular communication and cell signaling in the cytoplasm. Research performed by Frampton et al. and Li et al. revealed that restoration of the normal expression of miRNA-126 and miRNA-146a has been found to reverse EMT changes, restoring epithelial cell characteristics and reducing metastatic potential [160,161]. During metastasis formation, progressive reduction of miRNA-218 expression has been observed. miRNA-218 controls the expression of *ROBO1*, which functions as a regulator of proliferation [162,163,164]. Conversely, upregulation of miRNA-21 has been identified as a negative predictor of tumor progression by regulating PTEN and PDCD4 in PANC-1 and PA-TU-8988S cell lines [132]. Research on miRNA-4295 has revealed its role in metastasis development through interaction with GPC5 (Glypican 5) in the Wnt/β-catenin signaling pathway. GPC5 expression is downregulated in AsPC-1, Panc-1, BxPC-3 and SW1990 cell lines. Suppression of miRNA-4295 leads to upregulation of GPC5 expression, inhibiting cancer cell invasion. GPC5 acts as a negative regulator of the Wnt/β-catenin pathway by binding to Wnt3a [165].

### 3.5. Diagnostic Potential of miRNAs in Pancreatic Cancer

One of the principal reasons for the poor prognosis of pancreatic cancer is its late diagnosis. Clinicians often observe a lack of pronounced symptoms until the cancer has invaded surrounding tissues or metastasized to distant organs. Consequently, approximately 50% of patients seek medical attention when the cancer has already disseminated. Furthermore, the scarcity of sensitive and specific diagnostic biomarkers impedes early detection. The diagnosis of pancreatic cancer relies on imaging test and histopathological examination of the tumor tissue [1,2,9,16,17,18,19]. Although CA 19-9 is the most widely labeled antigen for distinguishing between benign and malignant lesions, it is inadequate for pancreatic cancer diagnosis due to its low sensitivity and specificity. CA 19-9 is also unsuitable for screening the general population due to the low prevalence of pancreatic cancer. This marker is mainly used to diagnose of pancreatic cancer recurrence after resection. CA 19-9 is a glycoprotein embedded in the cell membrane of various cells including those in the gastrointestinal tract and liver in fetal life, salivary glands, mature cells of the pancreas and bile ducts and bronchioles, as well as in semen or cervical mucus, thus CA 19-9 is detectable at low levels in healthy individuals. Only significantly elevated levels of CA 19-9 suggest the development of cancer elevated CA 19-9 levels are observed in most patients with advanced pancreatic cancer, but also in patients with other cancers (colorectal cancer, gastric cancer, gallbladder cancer, lung cancer) and non-cancerous conditions, such as such as gallstones, pancreatitis, cystic fibrosis and liver disease. Occasionally, the measurement of CA 15-3, CEA or CA-125 in the blood may aid diagnosis, but their levels are not specific for pancreatic cancer [5,6,7,8].

At least 50% of the thousands identified miRNAs are altered in tumors, according to global studies [166]. These studies have investigated miRNA expression in tissues and cells, as well as in extracellular fluids such as blood and saliva. miRNAs, small molecules transported in exosomes, are stable under varying pH and temperature conditions, making their profiles in blood and saliva reproducible and suitable for routine diagnostics [167]. Current advances in molecular biology, such as next-generation sequencing, microarrays and RT-qPCR using fluorescent probes, are used in routine diagnostic and are the hope for improvement in diagnostics.

Tumor tissue from PDAC patients showed overexpression of miRNAs: 196, 200a, 27a, 222, 210, 221, 155, 212 and decreased expression of miRNAs: 96, 146, 245, 122, 31, 34, 145 [168,169,170,171]. In blood, increased expression has been observed for miRNAs: 21, 196a, 155, 185, 191 [171,172]. This poses a challenge in distinguishing organ-specific molecules from those altered systemically. Another difficulty lies in standardizing miRNA expression and determining endogenous controls. Currently, no unique endogenous controls (housekeeping genes) have been designated, and expression is often determined by relative fold change compared to endogenous controls, complicating standardization. Expanded studies are needed to ascertain the potential role of these molecules in the diagnosis and treatment of pancreatic cancer.

The expression of miRNA in blood samples (serum or plasma) from healthy patients compared to a pancreatic cancer patients identified three potential miRNAs as early detection biomarkers: miRNA-22, miRNA-642b, miRNA-885-5p [40]. Abnormal expressions of: miRNA-21, miRNA-155, miRNA-196a, miRNA-210, miRNA-483-3p, miRNA-192 and miRNA-18a were detected in serum of patients diagnosed with PDAC relative to controls [172,173,174,175]. Despite researchers often focusing on other miRNAs than those already reported, elevated levels of miRNA-155 have been consistently documented in multiple independent studies [173,176,177]. Screening of 735 miRNAs in the blood of patients with pancreatic cancer identified miRNA-1290 as a potential biomarker with a sensitivity and specificity of approximately 80% which is comparable to the specificity of the CA 19-9 [178]. Studies on PDAC patients’ blood and early cancerous pancreatic tissues revealed overexpression of: miRNA-203b-5p, miRNA-342-5p, miRNA-337-5p, miRNA-149-5p, miRNA-877-5p, miRNA-203a-3p and decreased expression of: miRNA-1226-3p, miRNA-3182, miRNA-625-3p, miRNA-624-5p, miRNA-664a-5p. Of these, miRNA-1226-3p was selected as a potential biomarker [179]. Another diagnostic approach involves combining specific miRNA expression levels with CA-19-9 levels. A diagnostic panel including CA 19-9, miRNA-16 and miRNA-196a was effective in diagnosing pancreatic cancer [180]. Combining CA 19-9 and miRNA-27a-3p increased sensitivity to 85% and specificity to approximately 81% [181]. Studies on normal and cancerous pancreatic tissues demonstrated overexpression of miRNA-23a, miRNA-21, miRNA-155, miRNA-31, miRNA-100, miRNA-143, miRNA-2214 and downregulation of miRNA-148a, miRNA-375, miRNA-217 [182]. Additionally, overexpression of miRNA-196 combined with reduced expression of miRNA-217 could differentiate normal tissue from PDAC [183]. miRNA can also diagnose acute pancreatitis as lipase and amylase do not always reflect the disease state. Research on the blood acute pancreatitis patients showed changes in the expression of: miRNA-7, miRNA-9, miRNA-141, miRNA-122 compare to blood of healthy patients [184]. Saliva studies in pancreatic cancer patients revealed overexpression of miRNA-940 and downregulation of miRNA-3679, indicating the potential of miRNA expression determination as a non-invasive pancreatic cancer diagnostic marker [157]. Serum studies from pancreatic cancer patients and PANC-1 cell line showed significant upregulation of miRNA-23b-3 [52]. Certain miRNAs exhibit dysregulation not only in pancreatic tissue specifically, but also systemically like miRNA-21. However, most miRNAs show significant dysregulation specifically within tumor tissues (e.g., miRNA-245), blood samples (e.g., miRNA-196a), or metastatic cells (e.g., miRNA-4295). This specific dysregulation underscores the potential of miRNAs as promising diagnostic markers for various stages and aspects of cancer progression. Studies on the PANC-1 and MIA PaCa-2 cell lines revealed that the proapoptotic TP53TG1 gene is negatively regulated by miRNA-96. The TP53TG1 gene encodes a long non-coding RNA induced by p53 under cellular stress conditions [185]. The expression of various miRNAs in PDAC and acute pancreatitis is detailed in Table 3.

### 3.6. MiRNAs in the Prognosis and Treatment of Pancreatic Cancer

Pancreatic cancer is a highly lethal malignancy, with only a small fraction of patients benefiting from current treatments [9,10]. The primary treatment modality is tumor resection followed by chemotherapy. For patients with advanced-stage disease, neoadjuvant therapy followed by surgical resection is recommended, but only after confirming the presence of cancer. Gemcitabine was the first drug shown to improve median survival, albeit only by a few weeks [11,12]. Another commonly used chemotherapeutic agent, FOLFIRINOX (5-fluorouracil, irinotecan, oxaliplatin and leucovorin), in combination with gemcitabine was demonstrated to increase median survival to 11.1 months, as compared to 6.8 months with gemcitabine alone [12,20].

Recent studies have identified that decreased expression of miRNA-142-5p and increased miRNA-320c expression in PDAC patients correlate with resistance to gemcitabine treatment [13,14]. This highlights the potential of microRNAs to predict the efficacy of chemotherapeutic treatments. Conversely, high miRNA-142-5p expression has been associated with tumor sensitivity to gemcitabine [13]. According to study conducted by Yu et al. decreased miRNA-200c expression is linked with lower median survival [14]. A study involving 56 pancreatic cancer patients found a correlation between upregulation of miRNA-155, miRNA-203, miRNA-210 and miRNA-222 and a six-fold higher mortality [189]. Several studies have identifed miRNA-21 overexpression as a negative predictor of both tumor progression and treatment response [190,191,192]. In 2014, Wang et al. demonstrated that decreased levels of miRNA-124 was a negative prognostic factor [192].

miRNAs are not only suggested as prognostic factors for pancreatic cancer development and treatment response, but they also hold potential as anticancer therapeutics. It has been demonstrated that the application of mimetic miRNAs which are identical to the endogenous miRNAs, can sufficiently restore physiological functions. Another approach involves the use of miRNA antagonists, single-stranded oligonucleotides designed to bind single-stranded miRNA molecules, thereby downregulating specific oncogenic miRNAs [73].

According to a study conducted by Kent et al., the downregulation of miRNA-143/145 via adenovirus-mediated transfection in pancreatic cancer cell lines PANC-1 and MIA PaCa-2 inhibited cell growth and metastasis by suppressing the KRAS signaling pathway. Interestingly, the results indicate that the growth-inhibitory effect occurs only when both microRNAs exhibit reduced expression. Retroviral transfection of miR-143 and miR-145 targets KRAS and RREB1, establishing a feed-forward circuit that enhances Ras signaling in cell lines HPNE, MIA PaCa-2 and PANC-1. The proposed model describes a feed-forward regulatory circuit in which KRAS signaling, via RREB1, represses miR-143/145 transcription. This repression alleviates the negative regulation of KRAS and RREB1, thereby enhancing signaling through Ras effector pathways [67,73]. Restoration of normal miRNA-150 expression has been shown to reduce the proliferation of HPAF, Panc10.05 and Colo357 cells by targeting MUC4 [59]. According to a study by Ji Q et al., transfection of MiaPaCa2 and BxPC3 cells with a miRNA-34 mimic inhibited cell growth and enhanced the response to gemcitabine by targeting p53 [134]. Similarly, transfection of antisense miRNA-21 into: PANC-1, MIA PaCa-2, HS766T, SW1990, PL45, MPanc96 and Capan-1 resulted in reduction cell growth [48]. Weiss’s study demonstrated that transfection of pancreatic cancer cells with antisense miRNA-10a in zebrafish inhibited tumor growth [156]. In 2011, Rachagani et al. showed that transfection with antisense miRNA-132 and miRNA-212 led to decreased proliferation of PANC-1 cells [193].

## 4. Discussion

The involvement of miRNAs in various processes implicated in the pathogenesis of pancreatic cancer holds promise for their potential diagnostic and therapeutic applications. MiRNAs exert regulatory control over critical biological processes such as cell survival, proliferation, apoptosis and metastasis by targeting key molecules within numerous signaling pathways. While emerging evidence highlights the associations between dysregulated expression of specific miRNAs and various signaling pathways, their role in intercellular communication within pancreatic tumors remains incompletely understood. MiRNAs have been identified as dysregulated in pancreatic cancer tissues, as well as in serum and saliva samples of affected individuals, suggesting their potential as diagnostic or prognostic markers. Nonetheless, clinical studies exploring the diagnostic and prognostic capabilities of miRNAs in pancreatic cancer are limited in number. The detection of altered miRNA expression in serum samples offers a non-invasive approach to cancer diagnosis. Moreover, the potential of miRNAs to distinguish pancreatic cancer stages, predict treatment response and forecast survival outcomes requires further validation. Utilizing miRNAs in pancreatic cancer therapy holds promise, yet remains in its nascent stages of investigation preliminary findings from basic research indicate that restoring reduced levels of tumor suppressor miRNAs and inhibiting elevated levels of oncogenic miRNAs present compelling opportunities for developing novel and effective anticancer therapies. Thus, while significant strides have been made in understanding miRNA involvement in pancreatic cancer, translating this knowledge into clinical applications remains a burgeoning field.

## Figures and Tables

**Table 1:** Role of miRNA’s in the cell cycle and proliferation of pancreatic cancer.

miRNA	Expression	Target	Effect	Ref.
miRNA-21	↑	Reduction of *PTEN* expression	Increased cell proliferation	[48,132]
miRNA-424-5p	↑	Downregulation of SOCS6 expression. This leads to increased ERK pathway activity	Increased cell proliferation and migration	[119]
miRNA-124	↓	Negative regulation the expression of the Rac1 oncogene (MKK4-JNK-C-JUN) pathway	Activation tumor cell proliferation	[133]
miRNA-107	↓	Targeting *CDK6* encodes cyclin D1-dependent kinase	Activation of cell growth	[129]
miRNA-27a	↑	Negative regulation the expression of the Spry 2 suppressor gene	Activation tumour growth and migration	[134]
miRNA-143, miRNA-126	↑/↓ (Different)	Regulation of KRAS pathway expression.	Abnormal proliferations	[71,119,128]
miRNA-26a miRNA-223	↑/↓ (Different)	Regulation of cyclin E2 expression	Abnormal cell transition from G1 to S phase	[122,135]

**Table 2:** Apoptosis connected miRNA identified in pancreatic cancer.

miRNA	Expression	Target	Effect	Ref.
miRNA-34a	↓	Regulation of Bcl-2, Notch 1, Notch 2 expression	Avoidance of apoptosis	[143]
miRNA-155	↑	Suppression of the proapoptotic protein p53 (*TP53^INP1^* gene)	Inhibition of apoptosis	[150]
miRNA-23a	↑/↓ (Different)	Regulation the APAF1 factor	Promotion/Avoidance of apoptosis	[151]
miRNA-203	↓	Overexpression of survivin	Inhibition of apoptosis	[152,153]
miRNA-150 miRNA-603	↑/↓ (Different)	Regulate the expression of IGF-1R	Promotion/Inhibition of apoptosis	[145]
miRNA-196a miRNA-214	↑/↓ (Different)	Act on *TP53* via controlling the expression of ING4 and ING5	Promotion/Inhibition of apoptosis	[154,155]
miRNA-24	↑	Regulation of B1M expression, related to the BCL-2 family of proteins	Promotion of apoptosis	[146]

**Table 3:** Expression of various miRNAs in PDAC tissue compared to healthy pancreatic tissue and PDAC patients blood/saliva.

Sample Type	miRNA’s	Expression	Ref.
PDAC tissues	miRNA-196, miRNA-200a, miRNA-27a, miRNA-21, miRNA-222, miRNA-210, miRNA-221, miRNA-155, miRNA-212.	↑	[168,169,170]
	miRNA-200, miRNA-96, miRNA-21, miRNA-146, miRNA-245, miRNA-122, miRNA-31, miRNA-34, miRNA-145.	↓	
Blood of PDAC patients	miRNA-2, miRNA-18a, miRNA-21, miRNA-22, miRNA-24, miRNA-25, miRNA-99a, miRNA-155, miRNA-185, miRNA-191, miRNA-196a, miRNA-642b, miRNA-885-5p	↑	[171,172]
Saliva of PDAC patients	miRNA-940	↑	[157,186]
	miRNA-3679	↓	
Blood of acute pancreatitis patients	miRNA-7, miRNA-9, miRNA-122, miRNA-14	↑	[171,172]
PDAC migration and invasion tissues	miRNA-100, miRNA-21,miRNA-208, miRNA-194, miRNA-429, miRNA-200b, miRNA-200c	↑	[159,187,188]
	miRNA-10a, miRNA-34b, miRNA-143, miRNA-126, miRNA-146a, miRNA-31, Let-7, miRNA-4295, miRNA-218	↓	[156,160,161,162,163,164,165]

## Data Availability

No applicable.
